# Tear film thickness and stability following femtosecond-assisted laser *in situ* keratomileusis: a comparison of high- and low-myopia

**DOI:** 10.3389/fmed.2025.1650495

**Published:** 2025-09-24

**Authors:** Yan Li, Haibo Yan, Hua Yang, Shiyang Niu, Hong Qi, Chunyan Zhang

**Affiliations:** Department of Ophthalmology, The First Affiliated Hospital of Xinxiang Medical University, Weihui, Henan, China

**Keywords:** tear film thickness, first tear film breakup point, corneal epithelial thickness, tear film stability, femtosecond-assisted laser *in situ* keratomileusis

## Abstract

**Background:**

To investigate the relationship between tear film thickness, corneal epithelial thickness, tear film breakup location, and dry eye in patients with high- and low-myopia undergoing femtosecond-assisted laser *in situ* keratomileusis (FS-LASIK).

**Methods:**

A total of 52 patients (52 eyes) were enrolled and divided into a low-myopia group (LMG; spherical equivalent ≤−3.00 D; 26 eyes) and a high-myopia group (HMG; spherical equivalent ≥−6.00 D; 26 eyes). The Ocular Surface Disease Index (OSDI), fluorescein tear film breakup time (FBUT), corneal epithelial thickness (CET), tear film thickness (TFT), and other tear film stability markers were evaluated preoperatively. Each parameter was evaluated preoperatively at 1 and 3 months postoperatively.

**Results:**

At 1 month postoperatively, the HMG showed significantly higher OSDI scores and CET compared to the LMG (*p* < 0.05). Conversely, the FBUT and TFT were significantly lower in the HMG at the same time point (*p* < 0.05). Within a 6- to 7-mm corneal diameter, the TFT was significantly lower in the HMG than in the LMG (*p* < 0.05). Furthermore, the change in CET from baseline at 1 and 3 months postoperatively was significantly greater in the HMG, especially within the 5-mm corneal diameter (*p* < 0.05). There were no significant differences in spherical equivalent (SE) or uncorrected distance visual acuity (UDVA) between 1 and 3 months postoperatively within either group (*p* > 0.05).

**Conclusion:**

The observed alterations in tear film thickness, tear film distribution, FBUT, and tear film breakup location, affected by varying corneal stromal ablation depths, contribute to the development of dry eye disease following FS-LASIK. The extent of corneal epithelial remodeling after FS-LASIK correlates with the degree of refractive correction but not with refractive regression, and may play a role in tear film stability recovery.

## 1 Introduction

Dry eye disease (DED) is considered the most common complication of corneal refractive surgery (1, 2). It is the main cause of refractive regression, decline of visual quality, pain and discomfort, and a decrease in patient dissatisfaction, further affecting the work, lifestyle, and visual quality of patients (3–5).

The etiology of DED after corneal refractive surgery (CRS) has been reported in a large number of studies (6, 7), but the exact mechanism is still unclear. Neurotomy is a relatively recognized major cause of DED after CRS (8). Studies have shown that laser-assisted *in situ* keratomileusis (LASIK) causes severe corneal nerve injury due to the large corneal flap; 95% of patients exhibit dry eye symptoms immediately after undergoing LASIK (9), and 20–40% of dry eye symptoms can last until approximately 6 months postoperatively (10). However, studies of nerve recovery after CRS showed that at 1 year after LASIK, the subbasal nerve density was still lower than the preoperative level (11), but the corneal sensitivity had returned to a normal level (12). Therefore, the recovery times of nerve injury and corneal sensitivity after FS-LASIK surgery were inconsistent with those of DED. There may be other important factors neglected in the occurrence of DED after CRS.

The tear film is important to its underlying cornea and is closely related to prevalent DED (13). Abnormal tear film thickness or tear film distribution can cause DED (14). However, diseases with abnormal corneal morphology, such as corneal scarring or keratoconus, can cause changes in the distribution and stability of the tear film (15). The stability of the tear film is helpful to maintain the normal function of the corneal epithelium, and healthy corneal epithelium also plays an important role in maintaining the stability of the tear film (16). Corneal epithelial remodeling refers to the potential of the corneal epithelium to compensate for irregularities or changes of the underlying stromal surface shape by altering its thickness profile, which can maintain the integrity of the corneal optical surface (17–19). Further research is needed on corneal epithelial remodeling, including the corneal annular cutting area after FS-LASIK.

Therefore, the purpose of this study was to observe the relationship between tear film thickness, corneal epithelial thickness, the location of the first tear film break-up point, and dry eye in patients with high- and low-myopia after FS-LASIK surgery to improve the etiology theory of DED and to guide clinical treatment.

## 2 Patients and methods

### 2.1 Study design

This study was conducted from August 2023 to June 2024 at The First Affiliated Hospital of Xinxiang Medical University. Independent personnel are responsible for the confidential management of patients’ random grouping and grouping information, and examiners cannot obtain patients’ grouping information during data collection and analysis, to minimize the evaluation bias caused by examiners’ subjective factors.

### 2.2 Inclusion and exclusion criteria

Inclusion criteria (20): axial myopia, age between 18 and 40 years of age; a routine ophthalmic examination (with the exception of refractive error) with a stable refractive error and a minimum calculated residual corneal stromal bed thickness greater than 280 μm; no use of soft corneal contact lenses for more than 2-weeks; and ability to participate in follow-up. Additionally, their spherical refractive errors were required to range from 0 to −3.00 D and more than −6.00 D among myopia patients in the FS-LASIK group, and these patients had astigmatism of up to −2.00 D CYL.

Exclusion criteria: patients who suffered from external ophthalmic diseases or had undergone external ophthalmic surgery preoperation; patients who had received a preoperative tear embolism, and patients with unstable refractive error, progressive myopia or astigmatism, or any systemic disease that could affect wound healing (e.g., diabetes).

This study adhered to the tenets of the Declaration of Helsinki and was approved by the Ethics Committee of the First Affiliated Hospital of Xinxiang Medical University (the approval number: EC-024-601). All participants were fully aware of the purpose, procedures, and potential risks of the study and provided informed consent.

### 2.3 Myopia degree grouping

The study included 52 patients (52 eyes) divided into a low-myopia group (LMG; spherical equivalent ≤−3.00 D; 26 eyes) and a high-myopia group (HMG; spherical equivalent ≥−6.00 D; 26 eyes). The operations were performed by the same surgeon. General information about the study population is shown in Table 1.

**Table 1:** General information of the study population.

Parameter	HMG (*N* = 26)	LMG (*N* = 26)	*p*	Total (*N* = 52)
Age (y)	26.51 ± 4.75	28.58 ± 6.55	0.547	27.08 ± 5.71
NCT (mmHg)	14.34 ± 2.24	16.12 ± 1.90	0.193	14.71 ± 2.08
SEQ (D)	−7.30 ± 1.08	−2.75 ± 0.47	<0.001	−5.01 ± 2.38
UDVA (log MAR)	1.20 ± 0.31	0.66 ± 0.21	<0.001	0.92 ± 0.42
CDVA (log MAR)	0	0.00 ± 0.01	0.316	0.00 ± 0.01
LLT (nm)	77.42 ± 21.79	69.89 ± 20.71	0.331	73.62 ± 20.42
TMH (mm)	0.22 ± 0.03	0.22 ± 0.05	0.563	0.23 ± 0.06
NIKBUTf (s)	7.81 ± 4.21	7.85 ± 4.29	1	7.82 ± 4.56
NIKBUTav (s)	10.62 ± 4.23	10.51 ± 4.61	0.886	10.57 ± 4.52
SI (mm)	11.52 ± 8.73	11.18 ± 4.70	0.629	13.15 ± 4.36
OSDI	6.45 ± 1.38	5.56 ± 2.68	0.101	6.11 ± 2.52
FBUT (s)	7.26 ± 2.48	6.49 ± 1.89	0.277	6.86 ± 2.35
CFS	0	0	1	0.06 ± 2.02
Mean-CET (μm)	53.01 ± 1.96	53.00 ± 1.34	0.986	53.01 ± 1.06
Mean-TFT (μm)	28.13 ± 2.78	28.36 ± 3.56	0.783	28.25 ± 3.22

Data are presented as mean ± SD; LMG, low-myopia group; HMG, high-myopia group; mm means millimeter; μm, micrometer; nm, nanometer; s, seconds; SEQ, spherical equivalent refraction; D, diopters; UDVA, uncorrected distance visual acuity; log MAR, log of the minimum angle of resolution; CDVA, corrected distance visual acuity P, HMG vs. LMG; LLT, Lipid layer thickness; TMH, the tear meniscus height; NIKBUTf/NIKBUTav, non-invasive Keratograph first and average tear film breakup times; SI, Schirmer secretion test; OSDI, Ocular surface disease index; FBUT, Fluorescein tear film breakup time; CFS, cornea fluorescence staining score; CET, Corneal epithelial thickness; Mann–Whitney U test. *p* < 0.05 is considered to be a statistically significant difference.

### 2.4 Surgical protocol and medication

**Figure 1:**
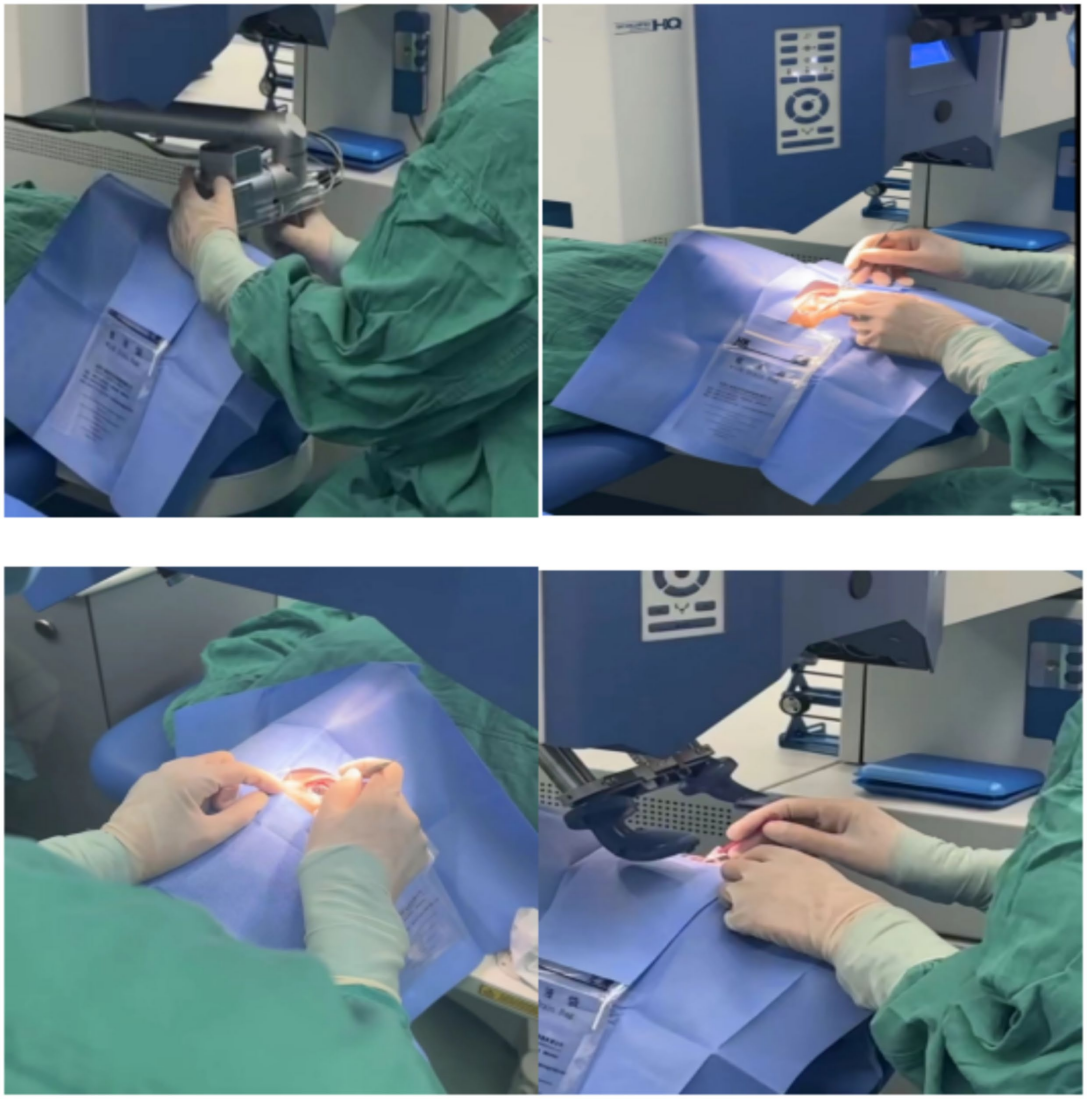
Surgical procedures used in this study. A femtosecond laser is used to make the corneal flap; open the corneal flap; flush the corneal flap; and reset the corneal flap.

The same surgeon performed all procedures. The FS-LASIK procedure used femtosecond laser equipment (FEMTO LDV Z2, Ziemer, Switzerland) to create a corneal flap with a thickness of 100 μm, a diameter of 8.5-mm, a hinge length of 4-mm, and a hinge position at 90°. Subsequently, an excimer laser device (AMARIS 500, Schwind, Germany) was used for refractive corneal stromal ablation, with an ablation frequency of 500 Hz and an effective optical ablation zone diameter of 6.3-mm. After ablation, the area was rinsed with saline to cleanse the ablated region and maintain corneal hydration before repositioning the corneal flap. Antibiotic and corticosteroid eye drops were then administered postoperatively (Figure 1).

After surgery, 0.3% ofloxacin eye drops (Tarivid; Santen, Inc., Tokyo, Japan) and 0.1% fluorometholone (Flumetholon; Santen, Inc.) were topically administered 4 times daily for 1-week, and artificial tears (sodium hyaluronate eye drops, URSAPHARM Arzneimittel GmbH, Germany) were applied 4 times per day for 2-weeks.

### 2.5 Assessment indicators

The examination was performed preoperatively and at 1 and 3 months postoperatively. Patients were required not to be exposed to any ophthalmic treatment or medication for 4 h before the examination. The order of examinations was as follows: spherical equivalent refraction (SEQ), uncorrected distance visual acuity (UDVA) and corrected distance visual acuity (CDVA; log of the minimum angle of resolution vision, logMAR), non-contact tonometer (NCT) measurements, and the following indicators. (1) Ocular surface disease index (OSDI): the OSDI is an internationally recognized index for evaluating the severity of ocular surface diseases; possible scores range from 0 to 100 (21). (2) Lipid layer thickness (LLT): A Lipiview Interferometer (TearScience Inc., Morrisville, NC) was used to measure the LLT. The LLT-ave was recorded. Credibility (conformance factor, CF) > 0.80 was required to ensure the accuracy of the data. (3) Keratograph 5 M: The non-invasive Keratograph first and average tear film breakup times (NIKBUTf/NIKBUTav), the tear meniscus height (TMH), and the location of the first tear film break-up point were recorded (22). (4) Fluorescein tear film breakup time (FBUT) and cornea fluorescence staining score (CFS): fluorescein sodium strips (containing 1.0 mg fluorescein sodium each) (Jingming New Technological Development Co., Ltd., Tianjin, China) were used to measure the breakup time. The CFS score was evaluated through a yellow filter using the Oxford scale. (5) Schirmer secretion test (SI): Without anesthesia, SI strips (Jingming New Technological Development Co., Ltd., Tianjin, China) were placed between the lateral and middle thirds of the lower eyelid, and the patient was asked to close their eye for 5 min. (6) Corneal epithelial thickness (CET): Using Fourier domain optical coherence tomography (FD-OCT) (RTVue XR AngioVue, Optovue Inc., Fremont, CA, United States) (23–25). All participants’ OCT scans were scheduled before 12:00 a.m. to reduce the influence of diurnal variations in CET. The system had an axial resolution of 5 μm and a scanning speed of 53,000 A-scans per second. It was ensured that all scans were centered on the hyperreflective corneal vertex reflex as the patient focused on the central fixation target (26). The CET within a 7-mm diameter was automatically recorded. (7) Tear film thickness (TFT): TFT was measured before any of the other examinations. The Pentacam (Oculus Company, Germany) system has high resolution, high accuracy, and repeatability (27, 28), and it can accurately locate the position of the corneal apex. The measurement was performed by the same technician in an examination room with a temperature of 24 °C and a relative humidity of 50% before 12:00 a.m. Corneal topography measurements were performed for patients’ natural pupils under scotopic conditions using the Pentacam HR. Only measurements marked as “OK” in quality specification were considered valid. A baseline corneal pachymetry map was derived from the topometric map. Then, 1 μL of 0.1% fluorescein in preservative-free balanced saline was softly instilled into the inferior cul-de-sac with a micropipette (14, 29). The subject was told to perform the scan again after several natural blinks. The built-in software of the Pentacam system was used to analyze and compare the corneal thickness map before and after fluorescein staining of each eye, and the TFT of each position within a 6-mm diameter of the cornea was automatically displayed. The examiner moved the mouse to select a 7-mm corneal diameter, with the grid line as a reference, and recorded the TFT.

### 2.6 Diagnostic criteria of dry eye

The diagnosis of DED was made using the standard diagnostic criteria published by the Asian Dry Eye Association in 2017: dry eye symptoms (OSDI ≥ 13) and an FBUT ≤5 s (30). If both eyes of the patients met the selection criteria, the right eye was uniformly selected as the detection eye.

### 2.7 Statistical analysis

The statistical analysis was performed using SPSS 23.0 for Microsoft Windows (Chicago, Illinois, United States). The sample size meets the statistical requirements. The results are expressed as the mean ± standard deviation (SD). Spearman’s correlation analysis was used. The Mann–Whitney U test and Kruskal-Wallis (K-W) test were used to test for abnormally distributed values. *p* < 0.05 was considered significant.

## 3 Results

### 3.1 Comparison of general information

The study included 52 participants (Conforming to the sample size range calculated by using PASS 11.0) with a mean age of 27.08 ± 5.71 years. There was no significant difference in the detection indices between the HMG and the LMG.

### 3.2 Comparison of pre- and postoperative indices

At 1 month postoperatively, SEQ, UDVA, CDVA, LLT, FBUT, and TFT were significantly decreased in both groups, and OSDI and CET were significantly increased (*p* < 0.05). At 3 months postoperatively, the CET values in both groups were significantly higher than those at 1 month postoperatively (*p* < 0.05); however, neither group showed a significant difference in SEQ or UDVA between 1 and 3 months postoperatively (*p* > 0.05) (Table 2).

**Table 2:** Changes between pre- and postoperative indices in the two groups.

Parameter	LMG (*N* = 26)			p*p*	HMG (*N* = 26)			*p*
	Preoperative	Postoperative	Postoperative		Preoperative	Postoperative	Postoperative	
		1 month	3 months			1 month	3 months	
SEQ (D)	−2.72 ± 0.42	−0.04 ± 0.41^*^	0.02 ± 0.38^#^	<0.001	−7.30 ± 1.08	−0.23 ± 0.31^*^	−0.21 ± 0.28^#^	<0.001
UDVA (logMAR)	0.65 ± 0.19	−0.08 ± 0.07^*^	−0.10 ± 0.06^#^	<0.001	1.20 ± 0.31	−0.10 ± 0.04^*^	−0.10 ± 0.08^#^	<0.001
CDVA (logMAR)	0.00 ± 0.01	−0.09 ± 0.05^*^	−0.10 ± 0.06^#^	<0.001	0	−0.10 ± 0.04^*^	−0.10 ± 0.08^#^	<0.001
LLT (nm)	69.89 ± 20.71	42.21 ± 17.56^*^	65.24 ± 23.17^‖^	<0.001	77.42 ± 21.79	44.87 ± 11.79^*^	72.43 ± 20.78^‖^	<0.001
TMH (mm)	0.22 ± 0.05	0.20 ± 0.03^*^	0.23 ± 0.03^‖^	0.025	0.22 ± 0.03	0.21 ± 0.03	0.24 ± 0.03^‖^	0.025
NIKBUT (s)	7.85 ± 4.29	5.47 ± 2.97^*^	9.23 ± 3.89^‖^	0.001	7.81 ± 4.21	4.20 ± 1.42^*^	8.71 ± 2.78^‖^	<0.001
NIKBUTav (s)	10.51 ± 4.61	8.61 ± 3.32^*^	12.67 ± 4.81^‖^	0.006	10.62 ± 4.23	6.43 ± 2.35^*^	11.52 ± 3.45^‖^	<0.001
SI (mm)	11.18 ± 4.70	7.71 ± 5.61^*^	12.86 ± 7.25	0.027	11.52 ± 8.73	7.13 ± 5.26	12.17 ± 6.98^#‖^	0.020
OSDI	5.65 ± 2.68	8.63 ± 4.21^*^	6.01 ± 2.31^‖^	<0.001	6.45 ± 1.38	10.78 ± 2.81^*^	6.98 ± 2.17^‖^	<0.001
FBUT (s)	6.49 ± 1.89	4.41 ± 1.38^*^	6.83 ± 2.31^‖^	<0.001	7.26 ± 2.48	3.53 ± 1.26^*^	6.36 ± 2.28^‖^	<0.001
CFS	0	0	0.04 ± 0.10	0.366	0	0	0	1
Mean-CET (μm)	53.00 ± 1.34	53.56 ± 2.12	55.49 ± 2.17^#‖^	<0.001	53.01 ± 1.96	54.83 ± 1.38^*^	57.45 ± 2.27^#‖^	<0.001
Mean-TFT (μm)	28.36 ± 3.56	22.59 ± 2.25^*^	29.17 ± 3.12^‖^	<0.001	28.13 ± 2.78	20.56 ± 3.19^*^	28.78 ± 2.59^‖^	<0.001

Data are presented as mean ± SD; LMG, Low-Myopia Group; HMG, High-Myopia Group. mm, millimeter; μm, micrometer; nm, nanometer; s, seconds; SEQ, spherical equivalent refraction; D, diopters; UDVA, uncorrected distance visual acuity; log MAR, log of the minimum angle of resolution; CDVA, corrected distance visual acuity P, HMG vs. LMG; LLT, Lipid layer thickness; TMH, the tear meniscus height; NIKBUTf/NIKBUTav, non-invasive Keratograph first and average tear film breakup times; SI, Schirmer secretion test; OSDI, Ocular surface disease index; FBUT, Fluorescein tear film breakup time; CFS, cornea fluorescence staining score; CET, Corneal epithelial thickness; Kruskal-Wallis (K-W test) test was used for inter-group test and Post hoc analysis. *p* < 0.05 is considered to be a statistically significant difference. ^*^Indicates a statistically significant difference between preoperative and 1 month postoperative with *p* < 0.05. ^#^Indicates a statistically significant difference between preoperative and 3 months postoperative with *p* < 0.05. ^‖^Indicates a statistically significant difference between 1 month postoperative and 3 months postoperative with *p* < 0.05.

### 3.3 Postoperative analysis of the measured indices

At 1 month postoperatively, the OSDI and CET were significantly higher in the HMG than in the LMG (*p* < 0.05); the opposite was true of the FBUT and mean TFT (*p* < 0.05). At 3 months postoperatively, the CET was significantly higher in the HMG than in the LMG (*p* < 0.05; Table 3). There was no significant difference in FBUT and TFT between the two groups (*p* > 0.05; Table 3).

**Table 3:** Postoperative analysis of the measured indices in two groups.

Parameter	1 month postoperative			3 months postoperative		
	LMG (*N* = 26)	HMG (*N* = 26)	*p*	LMG (*N* = 26)	HMG (*N* = 26)	*p*
SEQ (D)	−0.04 ± 0.41	−0.23 ± 0.31	0.061	0.02 ± 0.38	−0.21 ± 0.28	0.080
UDVA (logMAR)	−0.08 ± 0.07	−0.10 ± 0.04	0.963	−0.10 ± 0.06	−0.10 ± 0.08	0.816
LLT (nm)	42.21 ± 17.56	44.87 ± 11.79	0.168	65.24 ± 23.17	72.43 ± 20.78	0.325
TMH (mm)	0.20 ± 0.03	0.21 ± 0.03	0.341	0.23 ± 0.03	0.24 ± 0.03	0.237
NIKBUTf (s)	5.47 ± 2.97	4.20 ± 1.42	0.032	9.23 ± 3.89	8.71 ± 2.78	0.795
NIKBUTav (s)	8.61 ± 3.32	6.43 ± 2.35	0.005	12.67 ± 4.81	11.52 ± 3.45	0.420
SI (mm)	7.71 ± 5.61	7.13 ± 5.26	0.566	12.86 ± 7.25	12.17 ± 6.98	0.761
OSDI	8.63 ± 4.21	10.78 ± 2.81	0.047	6.01 ± 2.31	6.98 ± 2.17	0.107
FBUT (s)	4.41 ± 1.21	3.53 ± 1.26	0.013	6.83 ± 2.31	6.36 ± 2.28	0.546
CFS	0	0	1	0.04 ± 0.10	0	0.316
Mean-CET (μm)	53.56 ± 2.12	54.83 ± 1.38	0.041	55.49 ± 2.17	57.45 ± 2.27	0.004
Mean-TFT (μm)	22.59 ± 2.25	20.56 ± 3.19	0.012	29.17 ± 3.12	28.78 ± 2.59	0.388

Data are presented as mean ± SD; LMG, Low-Myopia Group; HMG, High-Myopia Group; mm, millimeter; μm, micrometer; nm, nanometer; s, seconds; SEQ, spherical equivalent refraction; D, diopters; UDVA, uncorrected distance visual acuity; log MAR, log of the minimum angle of resolution; CDVA, corrected distance visual acuity P, HMG vs. LMG; LLT, Lipid layer thickness; TMH, the tear meniscus height; NIKBUTf/NIKBUTav, non-invasive Keratograph first and average tear film breakup times; SI, Schirmer secretion test; OSDI, Ocular surface disease index; FBUT, Fluorescein tear film breakup time; CFS, cornea fluorescence staining score; CET, Corneal epithelial thickness P, HMG vs. LMG; Mann–Whitney U test. *p* < 0.05 is considered to be a statistically significant difference.

### 3.4 Comparison of the incidence of DED

**Figure 2:**
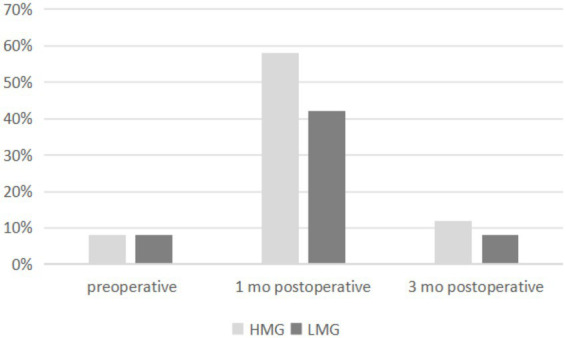
Distribution of prevalence of dry eye disease (DED) pre- and postoperatively in the high myopia group (HMG) and low myopia group (LMG).

**Figure 3:**
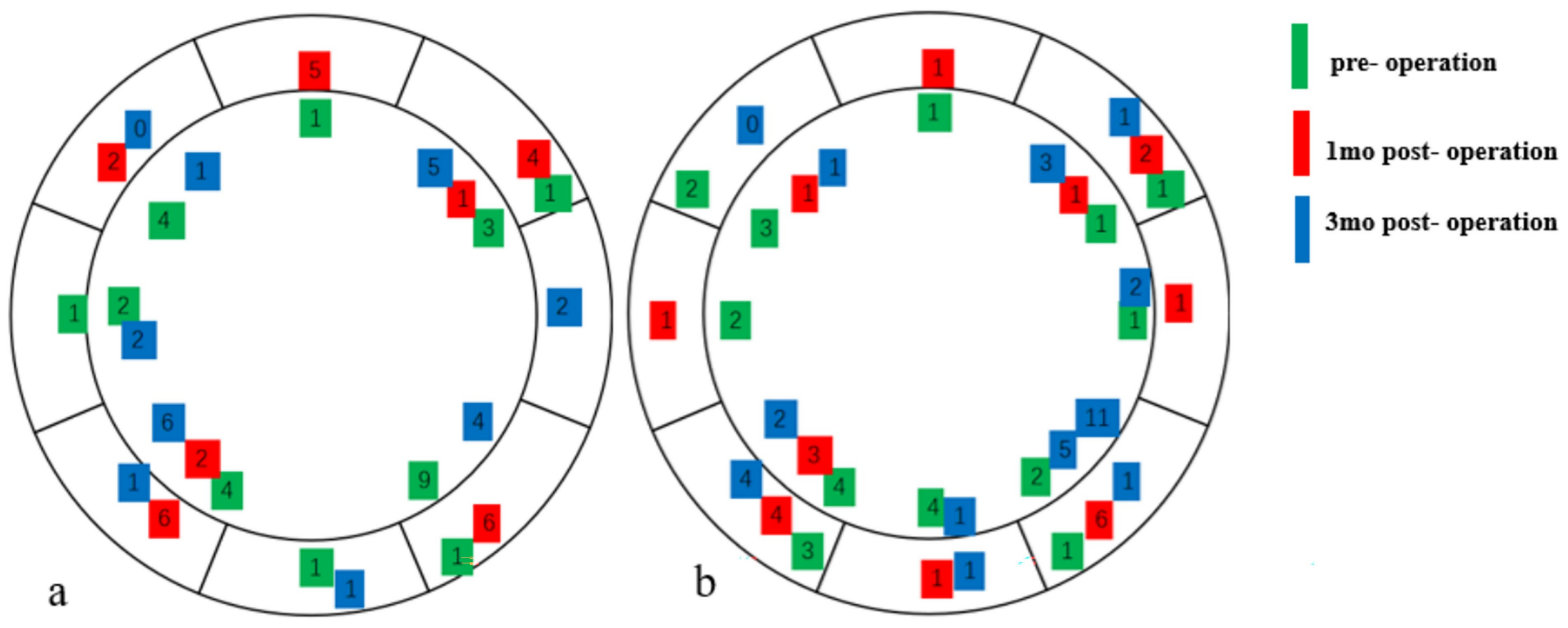
The location of the first tear film break-up points pre- and postoperative (Diameter range: ≤6 mm, 6–7 mm). **(a)** High-myopia group and **(b)** low-myopia group.

The prevalence of DED was 7% in both groups preoperatively, 59% in the HMG and 41% in the LMG at 1 month postoperatively, and 11% in the HMG and 7% in the LMG at 3 months postoperatively (Figure 2). The preoperative percentages of first tear film breakup points located within a 6- to 7-mm diameter of the cornea were 14% in the HMG and 28% in the LMG. At 1 month postoperative, the corresponding percentages were 87% in the HMG and 61% in the LMG (Figure 3). The percentages in both groups returned to the preoperative level at 3 months postoperatively.

### 3.5 Comparison of TFT

**Figure 4:**
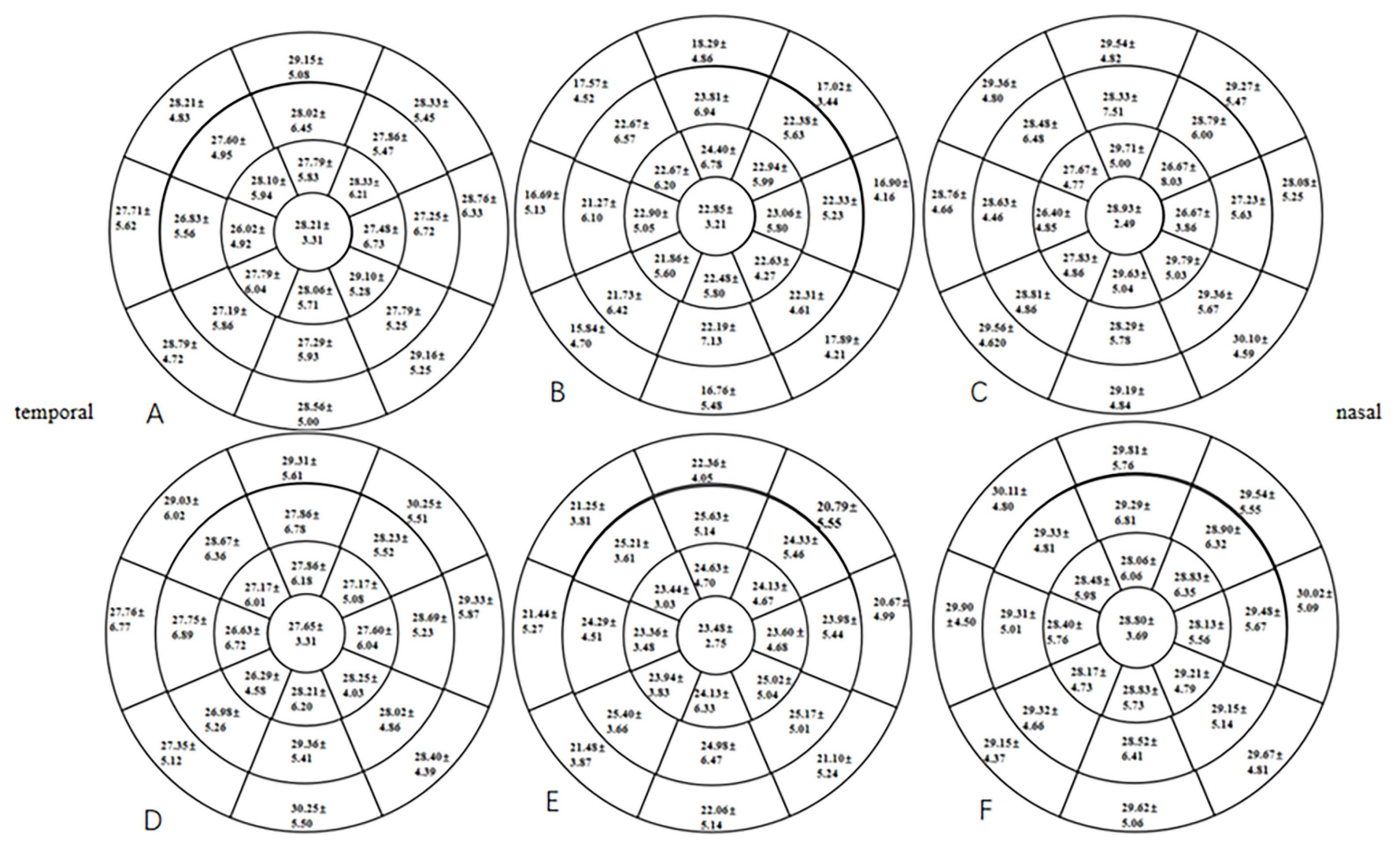
Distribution of TFT pre- and postoperatively in the HMG and the LMG (diameter range: 2, 4, 6, and 7 mm; unit of TFT: μm). **(A)** TFT in the HMG preoperatively. **(B)** TFT in the HMG at 1 month postoperative. **(C)** TFT in the HMG at 3 months postoperative. **(D)** TFT in the LMG preoperatively. **(E)** TFT in the LMG at 1 month postoperative. **(F)** TFT in the LMG at 3 months postoperative.

At 1 month postoperative, the mean TFT within a 7-mm diameter of the cornea in the two groups was significantly lower than the preoperative TFT (*p* < 0.05), especially in the HMG. The mean TFT within a 6- to 7-mm diameter of the cornea was significantly lower in the HMG than in the LMG (*p* < 0.05; Figure 4). The tear films of the two groups were evenly distributed preoperatively and at 3 months postoperatively, and the mean TFT within a 6-mm diameter of the cornea was not significant compared with that within a 6- to 7-mm diameter (*p* > 0.05).

### 3.6 Comparison of CET

**Figure 5:**
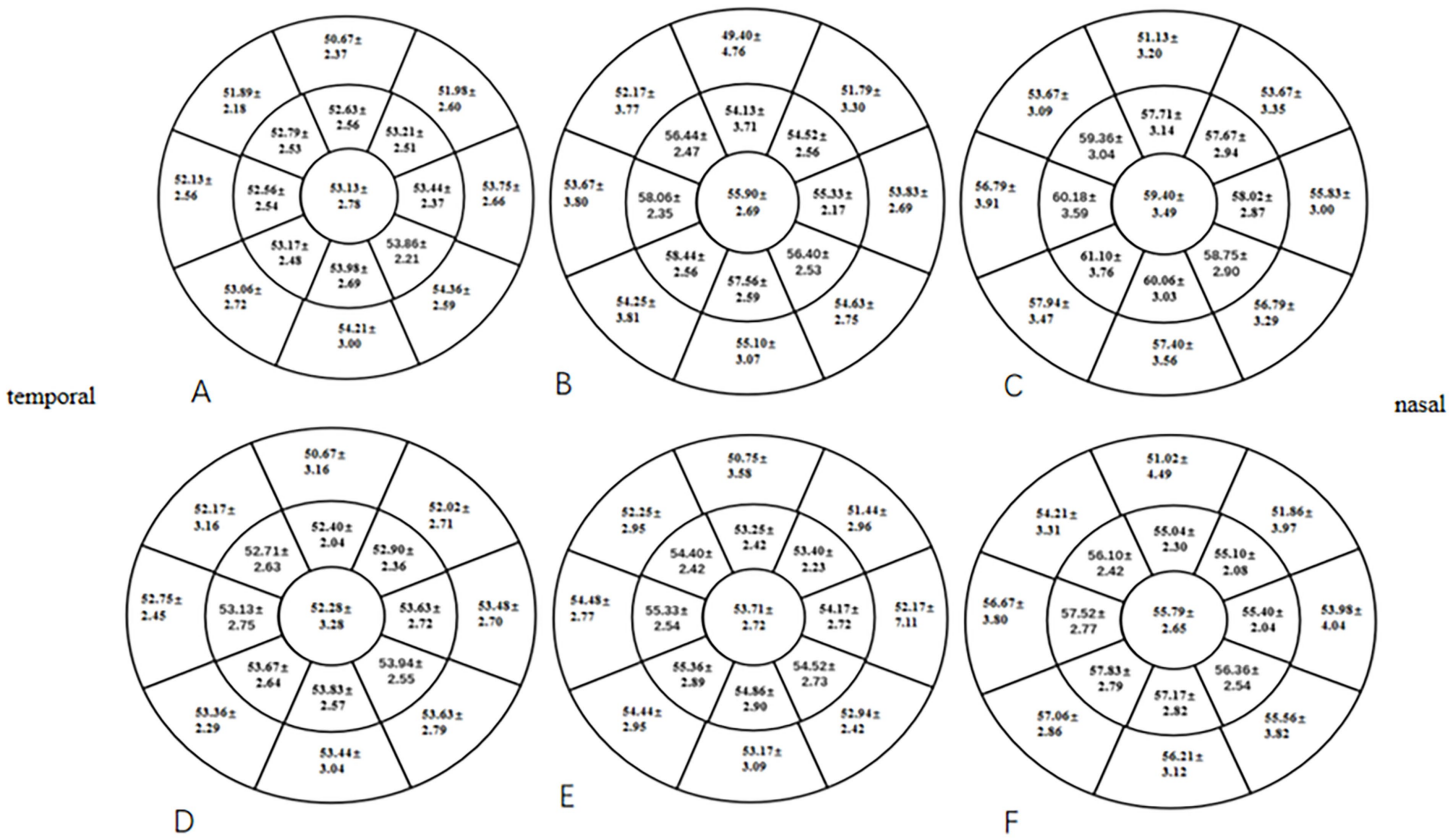
Distribution of CET pre- and postoperatively in the HMG and the LMG (diameter range: 2, 5, and 7 mm; unit of CEF: ìm). **(A)** CET in the HMG preoperatively. **(B)** CET in the HMG at 1 mo postoperative. **(C)** CET in the HMG at 3 mo postoperative. **(D)** CET in the LMG preoperatively. **(E)** CET in the LMG at 1 mo postoperative. **(F)** CET in the LMG at 3 mo postoperative.

**Figure 6:**
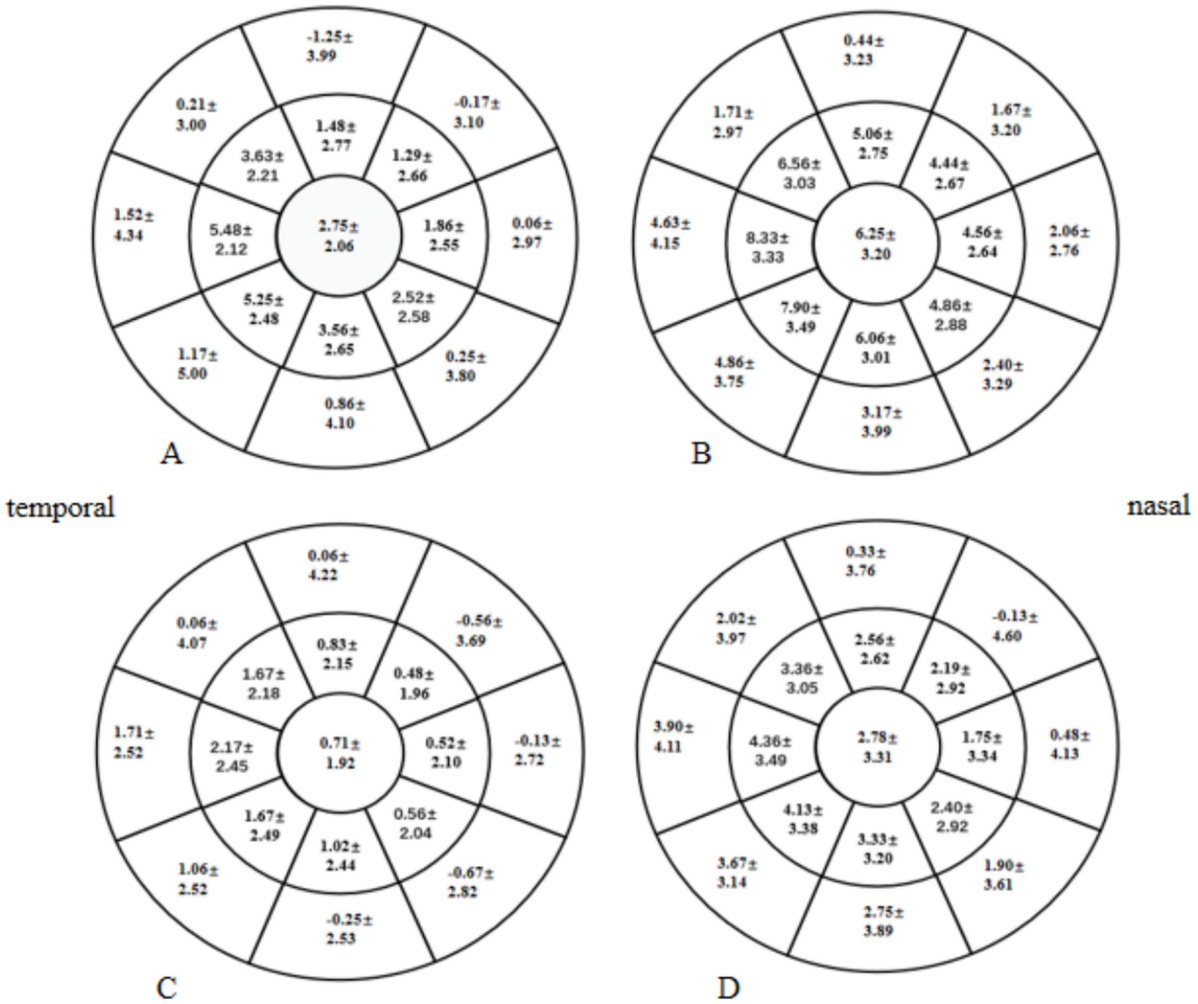
Distribution of postoperative increases in Corneal epithelial thickness (CET) in the high-myopia group (HMG) and low-myopia group (LMG) (diameter range: 2, 5, and 7 mm; unit of CET: μm). **(A)** Increase in CET in the HMG at 1 month postoperative vs. preoperatively. **(B)** Increase in CET in the HMG at 3 months postoperative vs. preoperatively. **(C)** Increase in CET in the LMG at 1 month postoperative vs. preoperatively. **(D)** Increase in CET in the LMG at 3 months postoperative vs. preoperatively.

At 1 and 3 months postoperative, the mean CET within a 7-mm diameter of the cornea in the two groups was significantly higher than the preoperative CET (*p* < 0.05), especially in the HMG (Figure 5). The added value of CET in the HMG was significantly higher than that in the LMG, especially in the 5-mm diameter of the cornea (*p* < 0.05; Figure 6).

### 3.7 Correlation analysis

**Figure 7:**
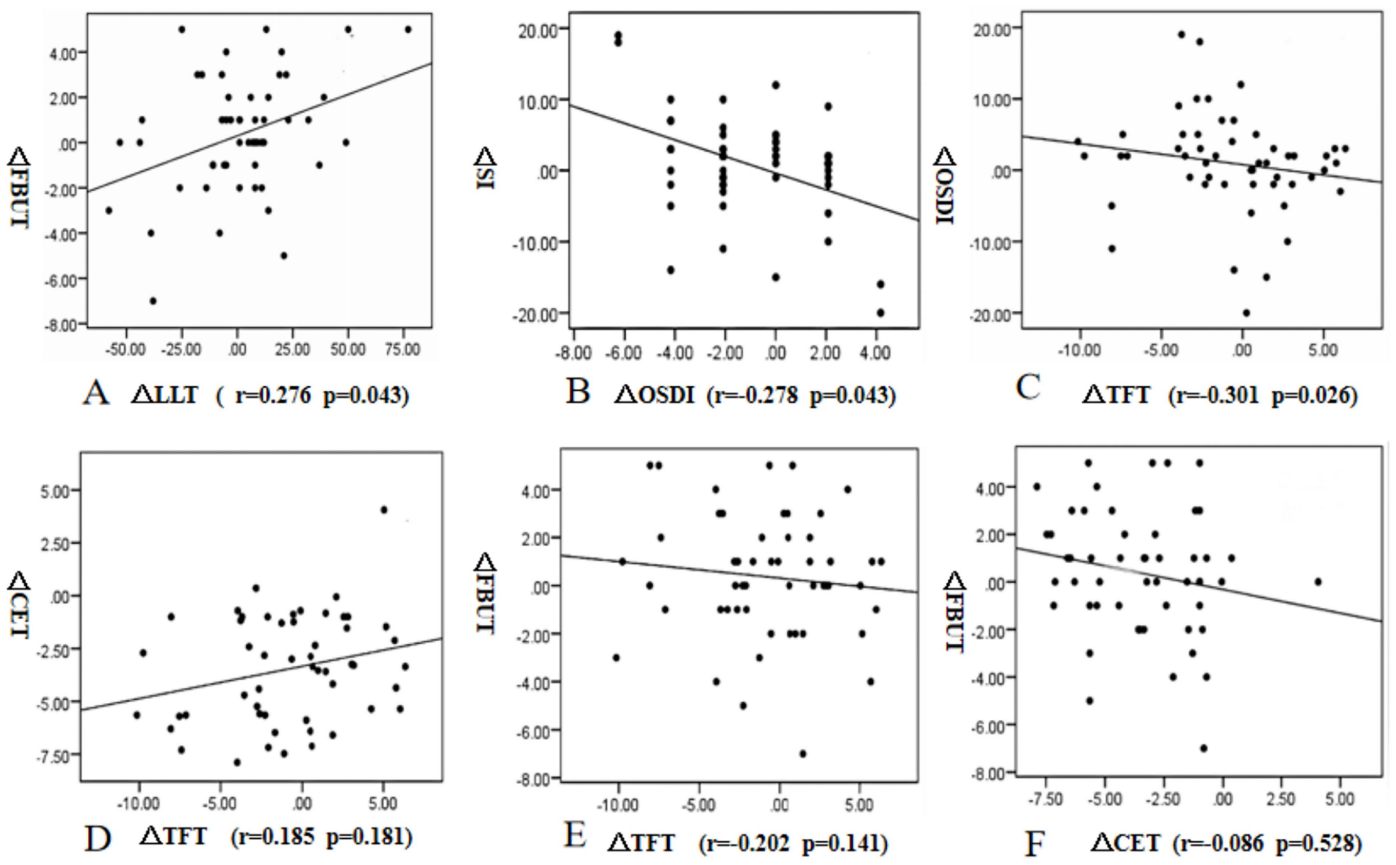
Correlations between the changes in lipid layer thickness (LLT) and fluorescein tear film breakup time (FBUT) **(A)**, the changes in Ocular surface disease index (OSDI) and Schirmer secretion test (SI), **(B)**, the changes in Tear film thickness (TFT) and Ocular surface disease index (OSDI). **(C)**, the changes in TFT and Corneal epithelial thickness (CET) **(D)**, the changes in TFT and FBUT **(E)**, and the changes in CET and FBUT **(F)** from preoperative to 3 months postoperative measurements.

In this study, the changes in values between preoperative and 3 months postoperative measurements were represented as delta indices. Correlation analysis was performed for changes at 3 months postoperatively. The change in LLT was significantly correlated with the changes in FBUT (r = 0.276, *p* = 0.043). The change in OSDI showed a negative correlation with the change in SI (r = −0.278, *p* = 0.043). The change in OSDI showed a negative correlation with the change in TFT (r = −0.301, *p* = 0.026). There were no correlations between the changes in CET and TFT (r = 0.185, *p* = 0.181), CET and FBUT (r = −0.202, *p* = 0.141), and TFT and FBUT (r = −0.086, *p* = 0.528) (Figure 7).

## 4 Discussion

In 2007, the International Dry Eye Workshop (DEWS) reported that the incidence of dry eye after laser *in situ* keratomileusis (LASIK) was 0.25–48.00% among patients without a history of dry eye (31). After femtosecond laser-assisted laser in situ keratomileusis (FS-LASIK), the tear film in patients with dry eye is unstable. Although the corrected vision is good, they still have blurred vision and vision fluctuation, which greatly affects the quality of life and work efficiency of patients (32). However, DED is still a common complaint among patients who undergo FS-LASIK surgery and could negatively affect the daily activities of patients (33).

Corneal nerves are densely distributed in the sub-basal layer of the corneal epithelium, and there are more than 7,000 nerve receptors per mm^2^ on the corneal surface. It is believed that corneal nerve fibers are disrupted during CRS, resulting in a decrease in corneal sensitivity, which leads to decreases in tear production and secretion by affecting the feedback loops that control those processes (34). We found that although the optical zone size of FS-LASIK surgery was the same in the HMG and the LMG, the prevalence of DED was different at 1 month postoperatively. Therefore, there may be other important causes for DED after FS-LASIK surgery.

The thickness and distribution of the tear film are closely related to DED (14). We found that 1 month after FS-LASIK, the TFT was thinner than that pre-operatively, and the incidence of dry eye increased. The incidence of dry eye decreased 3 months postoperatively, and the TFT basically returned to the preoperative level. Corneal shape abnormalities can affect the distribution of the tear film (35). The size of the optical zone of FS-LASIK patients included in this study was 6.3-mm; therefore, the changes in corneal shape and curvature at the annular edge of the cutting area (within a 6- to 7-mm diameter of the cornea) were obvious after FS-LASIK surgery. As the cutting depths of the corneal stromal layer were deeper in the HMG, the corneal curvature and shape tended to change more extensively at the annular edge of the cutting area. In this study, we used a Pentacam to acquire the distribution of the tear film (14). Keratograph 5 M was used to observe the variations in the location of the first tear film breakup point. We found that the TFT of the HMG in the annular edge of the cutting area of the cornea was significantly lower than that of the LMG at 1 month postoperative, and the percentage of the first tear film breakup points located in this annular region was significantly higher in the HMG than that in the LMG. At the same time, we found that the increase of CET in HMG was significantly higher than that in LMG at 1 month and 3 months after FS-LASIK. Studies have found that the steep corneal surface destroys the equilibrium state of surface tension between the tear film and corneal epithelium, causing abnormal tear film thickness and distribution, leading to a decrease in tear film stability (36). The area where the first tear film breakup point was located was the thinnest part of the tear film and the steepest part of the cornea, the location where the corneal curvature and shape change the most. It can be seen that early corneal shape changes after FS-LASIK surgery could cause changes in tear film thickness and distribution and changes in the location of the first tear film breakup point, resulting in DED.

Our results showed no significant difference in the tear film distribution, the location of the first tear film break-up point, and the prevalence of DED in the two groups at 3 months postoperative. They all recovered to preoperative levels. We inferred that with the passage of time, the function of each component of the tear film was restored, the tear film adapted to the changes in corneal shape and curvature, and the equilibrium state of the corneal epithelium was restored. Therefore, the tear film stability recovered. Correlation analysis showed that there was no correlation between the changes in TFT and FBUT. It may be related to the mean TFT used in our analysis. The influence of TFT on tear film stability is complex, the changes in tear film thickness, distribution, and components are all related to tear film stability.

The SI and TMH reflect the secretory function of the lacrimal glands. Consistent with the results of Kobashi et al. (37), they both changed slightly at 1 month after the procedure and returned to normal levels at 3 months postoperatively. Furthermore, there was no significant difference in SI and TMH between the HMG and the LMG postoperatively. These findings suggested that the effect of the operation on the corneal nerves was small, and tear production and secretion were unaffected. The effect of cutting depth on tear secretion function after FS-LASIK surgery was limited.

The OSDI represented the subjective scores of DED patients. Although the OSDI increased at 1 month postoperatively, it returned to its normal level at 3 months postoperatively. Studies have confirmed that the dry eye symptoms of the HMG were more serious than those of the LMG after corneal refractive surgery (CRS) (7, 38). Our results showed that at 1 month postoperatively, the TFT in the HMG was thinner and the tear film stability was lower than that in the LMG; therefore, the OSDI in the HMG was significantly higher than that in the LMG. Correlation analysis showed that the changes in TFT and OSDI were negatively correlated. It can be seen that the TFT has a certain influence on the subjective symptoms of patients.

FBUT is an important index that reflects the stability of the tear film. Consistent with the results found by Vestergaard et al. (39), we found that the FBUT values in both groups were significantly lower than the preoperative values at 1 month postoperatively. We also found that the FBUT in the HMG was lower than that in the LMG. Abnormal corneal shape affects the distribution and stability of the tear film. The cutting depth of the corneal stroma was deeper in the HMG; therefore, the corneal shape changed greatly (40), and the TFT decreased significantly, resulting in instability of the tear film. The TFT in both groups recovered to a normal level at 3 months postoperatively. Therefore, consistent with the results of Li et al. (33), the FBUT values in both groups returned to their preoperative levels.

CFS can be used to evaluate the changes in the corneal epithelium after CRS. It reflects the influence of CRS on corneal epithelial function. We found that there was no significant difference in CFS before and after FS-LASIK surgery; there was no significant difference in CFS between the HMG and the LMG. Though FS-LASIK surgery causes damage to the corneal nerve, there was no significant difference in the degrees of damage to the corneal nerve and epithelium caused by different cutting depths of the corneal stroma.

The lipid layer, located in the outermost layer of the tear film, helps the tear film redistribute after blinking and prevents water evaporation. Changes in its composition, distribution, and thickness are associated with DED (41). Our results showed that the LLT decreased significantly after FS-LASIK. Although the change in LLT was positively correlated with the change in FBUT, and the LLT in the HMG was higher than that in the LMG, the FBUT in the HMG was significantly lower than that in the LMG at 1 month postoperatively. The average thickness of the lipid layer was measured in our study. The measurement range of the Lipiview Interferometer (TearScience Inc., Morrisville, NC) was between the lower margin of the pupil and the lower eyelid margin. The thickness, composition, and distribution of the whole tear film lipid layer were not detected. Notably, studies have shown that although the thickness of the lipid layer has a certain protective effect on the stability of the tear film, the effect of the lipid layer on the tear film is complex. The quality, quantity, distribution, and composition of the lipid layer are all related to its function (42).

Corneal epithelial remodeling also occurred after CRS. Vestergaard et al. (39) and Reinstein et al. (43) used different methods to measure CET after FS-LASIK surgery, the results of which all showed that the CET increased significantly compared with its preoperative value. Our findings showed that the corneal epithelial thickening in HMG was significantly higher than that in LMG and was most significant within a 5-mm diameter of the cornea. The higher the degree of correction of refractive errors, the greater the thickening of the corneal epithelium (44). However, there was no significant difference in UDVA or SEQ between the two groups after FS-LASIK surgery. The tear film can fill the irregular interface between corneal epithelium, and a stable tear film helps to protect the normal structure and function of corneal epithelium, while healthy corneal epithelium plays an important role in maintaining the tension balance between corneal epithelium and tear film. Studies have found that tear film thickness and corneal epithelial thickness are interlinked, and the balance between these two layers plays an important role in maintaining normal visual quality (45). When the equilibrium state of the two layers is destroyed, the opposite distribution of the two layers is beneficial to form a smooth reflection plane. However, our results showed that the change in TFT has no correlation with the change in CET. It may be related to the mean value of CET and TFT within a 7-mm diameter of the cornea used in our analysis. Therefore, due to the influence of surface tension and surface morphology, the corneal epithelium will thicken in the area where the tear film is thinned (46). In addition, corneal epithelial cells have the function of remodeling to eliminate or reduce the swelling of the corneal anterior stroma surface (47), meaning that they also have the ability to remodel to stabilize the tear film. Studies have shown that corneal epithelial remodeling was essentially stable at 3 months after FS-LASIK surgery (48), consistent with the recovery time of TFT and FBUT. Therefore, corneal epithelial remodeling after FS-LASIK is not related to refractive regression; instead, it is beneficial to maintain the corneal shape and to maintain the equilibrium state of the surface tension between the tear film and corneal epithelium, which may be beneficial to the recovery of the thickness and uniform distribution of the tear film.

This study has some limitations. First, the use of the invasive tear film thickness measurement may have an amplification effect on tear film thickness. Second, our follow-up period is only 3 months after the operation. Third, the study on the changes of meibomian gland morphology and blinking mode is not reflected in this article, but the changes were analyzed in detail in another article.

## 5 Conclusion

In conclusion, although the sample size we included was limited and the follow-up time was short, we found that the thickness and distribution of tear film, FBUT and variations in the location of the first tear film break-up point were different for the different cutting depths of the corneal stromal layer; the degree of corneal epithelial remodeling after FS-LASIK was related to the degree of refractive correction, but not to refractive regression. It may promote the recovery of tear film stability.

## Data Availability

The datasets presented in this article are not readily available because none. Requests to access the datasets should be directed to YL, yanlihn@yeah.net.
